# An Integrated Approach Using Spatial Analysis to Study the Risk Factors for Leishmaniasis in Area of Recent Transmission

**DOI:** 10.1155/2015/621854

**Published:** 2015-07-01

**Authors:** Júlia Alves Menezes, Eduardo de Castro Ferreira, José Dilermando Andrade-Filho, Alessandra Mara de Sousa, Mayron Henrique Gomes Morais, Ana Maria Sampaio Rocha, George Luis Lins Machado-Coelho, Fernanda Pinheiro Lima, Ana Paula Madureira, Tânia Cristina Garcia, Christian Resende Freitas, Rodrigo Pedro Soares, Carina Margonari

**Affiliations:** ^1^Centro de Pesquisas René Rachou, Avenida Augusto de Lima 1715, 30190-002 Belo Horizonte, MG, Brazil; ^2^Fundação Oswaldo Cruz Mato Grosso do Sul, Rua Gabriel Abrão, 79081-746 Campo Grande, MS, Brazil; ^3^Fundação Educacional de Divinópolis, Universidade do Estado de Minas Gerais, Avenida Paraná 3001, 35501-170 Divinópolis, MG, Brazil; ^4^Universidade Federal de Ouro Preto, Campus Universitário Morro do Cruzeiro, 35400-000 Ouro Preto, MG, Brazil; ^5^Centro Universitário de Formiga, Avenida Dr. Arnaldo de Senna 328, 35570-000 Formiga, MG, Brazil; ^6^Universidade Federal de São João Del Rei, Praça Dom Helvécio 74, 36307-352 São João Del Rei, MG, Brazil; ^7^Instituto de Geociências, Universidade Federal de Minas Gerais, Avenida Antônio Carlos 6627, 31270-901 Belo Horizonte, MG, Brazil

## Abstract

Some epidemiological aspects of leishmaniasis in the municipality of Formiga, Brazil, an important touristic site, were evaluated. Those included phlebotomine sand fly vectors, canine infection, and geoprocessing analysis for determining critical transmission areas. Sand flies (224 insects) belonging to ten different species were captured. The most captured species included *Lutzomyia longipalpis* (35.3%), *Lutzomyia cortelezzii* (33.5%), and *Lutzomyia whitmani* (18.3%). A significant correlation between sand fly densities and climatic conditions was detected. Serological diagnosis (DPP and ELISA) was performed in 570 dogs indicating a prevalence of 5.8%. After sequencing the main species circulating in the area were *Leishmania infantum* and *Leishmania braziliensis*. Spatial analysis demonstrated that vegetation and hydrography may be related to sand fly distribution and infected dogs. The municipality of Formiga has proven leishmaniasis vectors and infected dogs indicating the circulation of the parasite in the city. Correlation of those data with environmental and human cases has identified the critical areas for control interventions (south, northeast, and northwest). In conclusion, there is current transmission of visceral and canine human cases and the city is on the risk for the appearance of cutaneous cases.

## 1. Introduction

Leishmaniases are a spectrum of diseases caused by intracellular protozoan parasites from the genus* Leishmania* (Kinetoplastida: Trypanosomatidae). They are transmitted to the vertebrate host by phlebotomine sand flies (Psychodidae: Phlebotominae) during the bite. Their clinical manifestations may range from self-healing cutaneous to fatal, if untreated, visceral form [1]. They are endemic in 98 countries and considered by the World Health Organization one of the most important neglected diseases [2]. Despite being a rural disease, VL is urbanizing because of environmental changes and vector adaptation to human habitats and domestic reservoirs [3]. In Brazil, cutaneous (CL) and visceral forms (VL) are a very important public health problem, where an incidence of 30,000 and 3,400 new cases/year is reported, respectively [2, 4]. Although control measures exist, an intense geographic expansion of the disease in the past decades was observed, especially for VL in many major Brazilian cities [5–8].

Consistent with those observations, in Minas Gerais state, despite the occurrence of rural human cases, many municipalities are reporting urban cases, including the state's capital, Belo Horizonte, considered endemic for VL [9–12]. In the state, many studies in different regions have demonstrated the occurrence of proven vectors of VL (*Lutzomyia longipalpis*) and CL (*Lutzomyia whitmani* and* Lutzomyia intermedia*) [13–16]. In addition, the abundant presence of synanthropic/domestic infected reservoirs (marsupials, rodents, and canids) reinforces the role of Minas Gerais as one of the most important transmission areas in the country [17–21].

**Figure 1:**
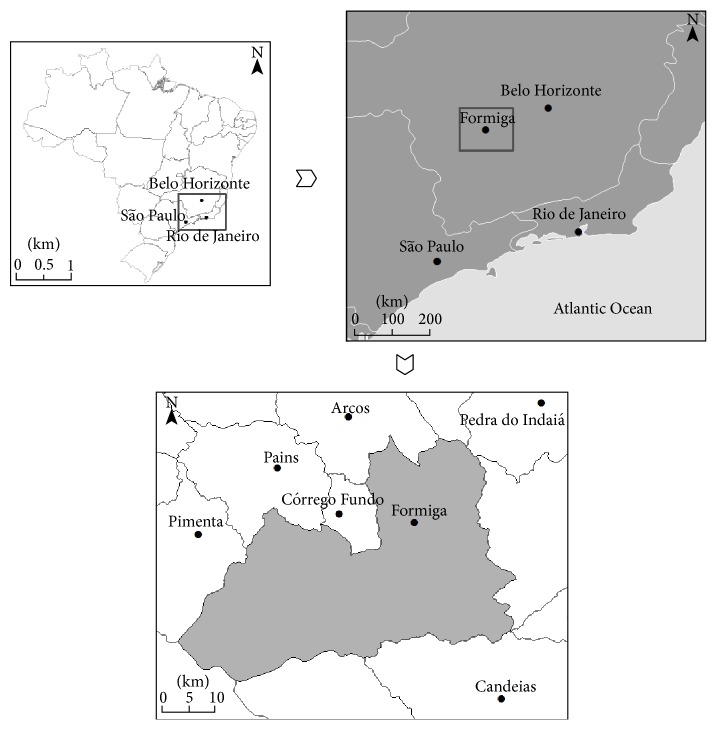
Localization of Formiga in Minas Gerais state, Brazil.

Formiga city (Figure 1) is considered a recent transmission area for leishmaniasis, according to the Ministry of Health (average number of cases ranging from 0 to 2.4 in the past 5 years). The first VL cases started to occur after 2011 resulting in two deaths, bringing interest to the health authorities since the disease had never been detected previously. The municipality of Formiga is part of a touristic complex called Furnas Lake, a hydroelectric dam responsible for one of the largest freshwater reservoirs of Brazil.

Geoprocessing techniques can help to understand how the determinants of disease transmission are related, linking ecological and sociodemographic factors with the incidence and distribution of a given disease. In the case of leishmaniasis, geographic information systems (GIS) found associations between epidemiological components of the disease (sand flies, human and canine cases) and environmental features (vegetation, sanitation, and altitude) [22–25]. However, the transmission profile of the disease can be highly variable depending on the region. Those aspects are completely unknown for the city of Formiga. This city is in an area where leishmaniasis cases were detected in other surrounding cities [12, 16, 26, 27]. This work aimed to (1) study the sand fly vectors fluctuations and (2) dog infection and (3) to perform a spatial analysis of the factors studied to identify the critical areas in the city.

## 2. Material and Methods

### 2.1. Ethical Statement

This project was approved in accordance with Internal Ethics Committee in Animal Experimentation (CEUA) of Fundação Oswaldo Cruz (FIOCRUZ), Belo Horizonte, Minas Gerais, Brazil (Protocol p-0297-06).

### 2.2. Study Area

The municipality of Formiga (20° 27′ 52′′ S and 45° 25′ 35′′ W) is located in the central-western region of Minas Gerais state (~120 km from the capital's state) and has 65,128 inhabitants in an area of 1502 km^2^ (Figure 1). Its altitudes range from 785 to 1,125 m. The vegetation is a transition between Cerrado and Atlantic Forest under a tropical climate. Annual average temperatures and precipitations are 21.8°C and 1272 mm, respectively [28].

### 2.3. Sand Flies

#### 2.3.1. Systematic Collections, Species Identification, and Weather Conditions

**Figure 2:**
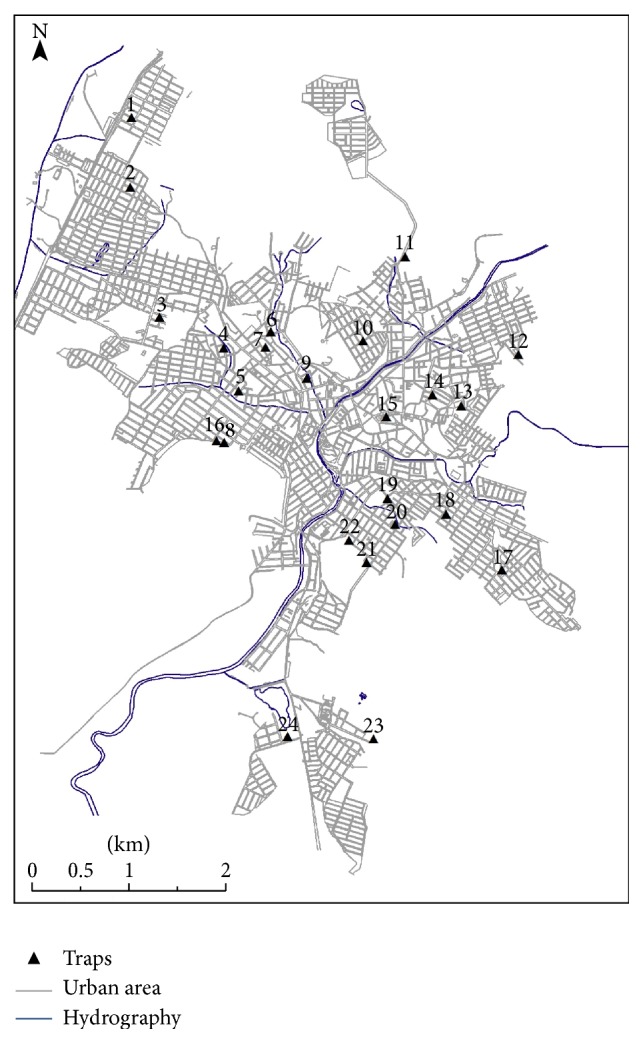
Location and distribution of HP light traps throughout the urban area of Formiga, Minas Gerais state, Brazil.

Captures were performed between May/2012 and April/2013 during four consecutive days per month. HP traps [29] were used in the peridomiciliary areas of 24 houses from 5 pm to 7 am. Traps distribution followed information provided by the health authorities according to the occurrence of human and canine leishmaniasis cases, resulting in 22 preferential regions for collection. Within these regions, 24 houses were selected by convenience, in accordance with the environmental features of the domicile (backyards, trees, domestic animals, proximity to vacant lots, and green spots) (Figure 2). Captured insects were mounted [30] before taxonomic identification [31]. Temperature, relative humidity, and precipitation data were obtained from Formiga Meteorology Station (A524) during the collection period.

### 2.4. Canine Infection

#### 2.4.1. Sample Collections, Clinical Evaluation, and Serology Tests

The canine sample was calculated considering the World Health Organization parameters [32]. Since no previous information on prevalence was available, WHO recommends considering it as 50%. The estimated number of dogs in city (followed the antirabies campaigns) was 9,000. Based on that information, sample calculation generated a minimum sample of 517 dogs (confidence level was 90% and a sample error of 3.5%). A higher number (570) of male and female domestic mongrel dogs were evaluated. Peripheral blood samples (3–5 mL) and clinical evaluations were performed in the Center of Animal Life Defense (CODEVIDA) and in five veterinary clinics. Blood samples were stored in ice and sent to the Laboratório de Epidemiologia das Doenças Parasitárias of Universidade Federal de Ouro Preto (LEPI/UFOP) for antibody detection using serology tests. Dual Path Plataform (DPP), a rapid immunochromatographic test, and Enzyme Linked Immunosorbent Assay (ELISA) were used for canine leishmaniasis diagnosis as recommended by the Brazilian Ministry of Health. Only positive dogs in both serological tests were considered infected. The clinical features of the dogs were evaluated by a veterinarian doctor, who determined the physical characteristics and symptoms of the disease.

#### 2.4.2. Molecular Identification of* Leishmania*

To identify* Leishmania* species, DNA was extracted from the peripheral blood samples [15, 16] with subsequent amplification of the SSUrRNA gene by nested PCR (LnPCR). The SSUrRNA is a gene encoding the small subunit of the ribosome and is well conserved among the species of the genus* Leishmania*. For the LnPCR technique, specific primers for the order Kinetoplastida (R221 and R332) were used resulting in a 603 bp fragment. Then, a second reaction was performed with* Leishmania*-specific primers (R223 and R333) to obtain a 353 bp fragment. LnPCR reactions and thermal profile were as described elsewhere [33] with modifications [34]. Amplified PCR products were visualized on agarose gels (1.5%) stained with ethidium bromide.

The polymerase chain reaction-restriction fragment length polymorphism (PCR-RFLP) was also used to confirm* Leishmania* species by digestion of a 120-bp fragment of the kinetoplast minicircle conserved region with* Hae*III [16, 35]. The restriction profile was visualized on agarose gels (4%). Reference strains of* Leishmania (Leishmania) infantum* (MHOM/BR/74/PP75),* Leishmania (Viannia) braziliensis* (MHOM/BR/75/M2903), and* Leishmania (Leishmania) amazonensis* (IFLA/BR/67/PH8) were used as controls [16].

For sequencing, LnPCR fragments were extracted from the agarose gel using QIAquick PCR Purification kit (QIAGEN). The sequencing was performed using BigDye Terminator v3.1 CycleSequencing kit according to the manufacturer's specifications. The sequences were determined by DNA sequencer ABI3730 (Life Technologies) and analyzed using the BioEdit program for editing and alignment. The sequences were compared to those existing in GenBank database to identify* Leishmania* species.

### 2.5. Geoprocessing

#### 2.5.1. Maps Elaboration

The sand fly density map was elaborated based on the collection points and the number of captured insects. The data were georeferenced using the coordinates UTM SAD69 Fuso 23 determined by GPS (N3 Elgin). Seropositive dogs were georeferenced using the home addresses following the same methodology.

Kernel density maps [16] were elaborated using the Spatial Analyst tool from the software ArcGis 10. This method calculates the ratio between the number of sandflies/dogs and a given unit area. It calculates the average of dogs/sandflies occurrences separately per unit area. For this study, the unit area was defined in m^2^. Influence areas of 1 km radius for sand flies and 200 m radius for domestic dogs were established around each house on the map. Such approach enabled the visualization of sand fly influence based on the flight mean distance of those insects. On the other hand, the dogs included in this study were domestic, and for this reason they are not likely to circulate outside the houses. The density results were stored in a type raster file in the format GRID. Ten-meter pixels were set as spatial resolution using 1 : 100,000 scale.

#### 2.5.2. Signature Analysis

The signature method [22, 36] was used to identify the correlations among environmental features, vectors, and infected dogs using the Arcview software. This method consists in the definition of a variable of interest (density of infected dogs and sand flies) and the subsequent crossing of this with the other variables analyzed in the study (environmental features). The environmental variables are collected from official cartographic bases (rivers, urban areas) and subproducts of image classification (green areas). The maps are obtained from satellite images that do not differentiate vegetation types that may include Atlantic forest, savannah, and Cerrado or green areas (e.g., parks). Water areas include rivers, creeks, lakes, dams, and ponds. Then, the data are converted in raster format followed by the elaboration of a distance map between the environmental variables occurrence and the remaining of the analyzed territory. The objective is to know how close are the areas with higher dog infection rates in relation to traps, rivers, green, and urban areas. All information for the procedure was modeled in raster format, with extension and pixel sizes compatible with each other (10 m^2^). Crossed variables were total sand fly density,* L. longipalpis* density,* L. whitmani* density, infected dogs density, hydrography, and vegetation.

### 2.6. Statistical Analysis

Sand fly collection data were analyzed using GraphPad Prism 5.0 software (GraphPad Prism Inc., San Diego, CA). D'Agostino-Pearson normality test was performed to test the null hypothesis that the data were sampled from a Gaussian distribution [37]. When the data deviated from a Gaussian distribution, Spearman's correlation coefficient was used (significance was taken for *p* < 0.05 for a two-tailed test).

## 3. Results

### 3.1. Phlebotomine Sand Fly Fauna

Two hundred and twenty-four insects (151 males and 73 females) belonging to 10 species were collected including:* Lutzomyia longipalpis* (Lutz and Neiva 1912),* Lutzomyia cortelezzii* (Brethés 1923),* Lutzomyia whitmani* (Antunes and Coutinho 1939),* Lutzomyia lenti* (Mangabeira 1938),* Lutzomyia sordellii* (Shannon and Del Ponte 1927),* Lutzomyia monticola* (Costa Lima 1932),* Lutzomyia lutziana* (Costa Lima 1932),* Lutzomyia bacula* (Martins, Falcão, and Silva, 1965),* Lutzomyia brasiliensis* (Costa Lima 1932), and* Lutzomyia termitophila* (Martins, Falcao and Silva, 1964). Higher frequencies of captured sand flies were observed for* L. longipalpis* (35.3%) followed by* L. cortelezzii* (33.5%),* L. whitmani* (18.3%), and* L. lenti* (7.9%) (Table 1). Based on the index of positive sites (IPS = number of positive traps/total number of traps),* L. cortelezzii* and* L. longipalpis* were the most dispersed species. They were found in 92% and 75% of the houses, respectively. On the other hand,* L. whitmani* was found only in four sites, where two traps captured 90% of the sand flies.

**Figure 3:**
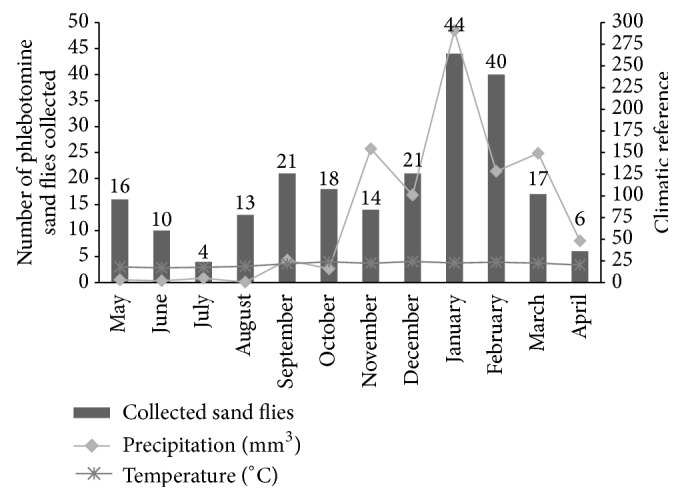
Climatic conditions affecting sand fly occurrence in Formiga. Temperature (°C), precipitation (mm^3^), and sand flies captured with HP traps between May 2012 and April 2013 in Formiga, MG, Brazil.

Significant correlations were observed between climatic conditions and sand flies abundance. A high correlation (*r* = 0.72; *p* = 0.008; *ρ* ^2^ = 0.52) was noticed between precipitation and sand flies occurrence. A moderate correlation was detected with temperature (*r* = 0.60; *p* = 0.039; *ρ* ^2^ = 0.36). No correlation was found with humidity (*r* = 0.09; *p* = 0.77). Consistent with those observations, following Qui-squared test, 52% and 36% of sand fly fluctuations could be explained by variations in precipitation and temperature, respectively. Most of sand fly captures (37.5%) occurred during summer months (January and February), where higher temperatures and precipitations were present (Figure 3).

### 3.2. Dog Infection, Clinical Evaluation, and Species Identification

**Figure 4:**
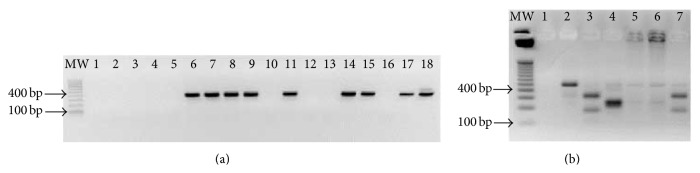
Molecular detection and identification of* Leishmania*. (a) Nested PCR technique (LnPCR). Agarose gel (1.5%) of the second reaction of the LnPCR for the 353 bp fragment. Lanes: MW; 100 bp-ladder; 1 and 12, negative controls; 18, positive control; positive samples (6–9; 11; 14-15; and 17); negative samples (2–5; 10; 13; and 16). (b) Restriction fragment length polymorphisms (RFLP-PCR) of positive canine samples in the LnPCR. Agarose gel (4%) of PCR-RFPL fragments after enzymatic digestion with* HaeIII*. Lanes: MW, 100-bp ladder; 1, negative control; 2,* L. amazonensis* (IFLA/BR/67/PH8); 3,* L. braziliensis* (MHOM/BR/75/M2903); 4,* L. infantum* (MHOM/BR/74/PP75); 5 and 6, dogs infected with* L. infantum*, and 7, dog infected with* L. braziliensis*.

Five hundred and seventy male and female domestic mongrel dogs were evaluated by serological techniques and LnPCR. Considering the positivity in both DPP and ELISA and the two serological methods required by the Brazilian Ministry of Health, the canine seroprevalence was 5.8% (*n* = 29). LnPCR technique detected 45 positive dogs (7.9%) and from those 18 was also seropositive. A representation of LnPCR and PCR-RFLP is shown in Figures 4(a) and 4(b). PCR-RFLP identified 19 positive samples. From those, only 13 could be identified after sequencing, where 10 dogs were infected with* L. infantum* and 3 with* L. braziliensis*. Those data indicate the presence of both species in domestic dogs from Formiga. Thirty-eight percent of the seropositive dogs (*n* = 11) were subclinically infected.

### 3.3. Spatial Analysis and Geoprocessing

**Figure 5:**
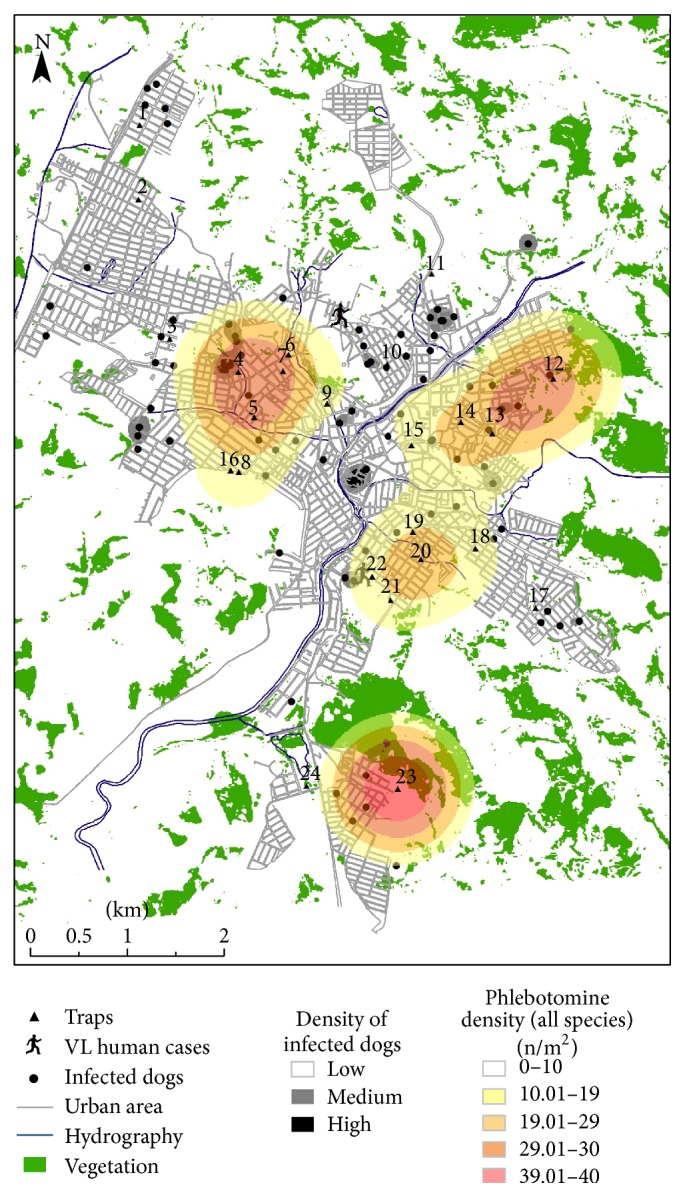
Geoprocessing analysis of total captured sand flies and parameters studied. Kernel density map of captured sandflies, canine VL cases, and human VL cases (human icon) and representation of vegetation and hydrographic variables in the urban area of Formiga, Minas Gerais, Brazil. Dark circles indicate positive dogs either by serological or molecular methods. n, number of collected sand flies.

**Figure 6:**
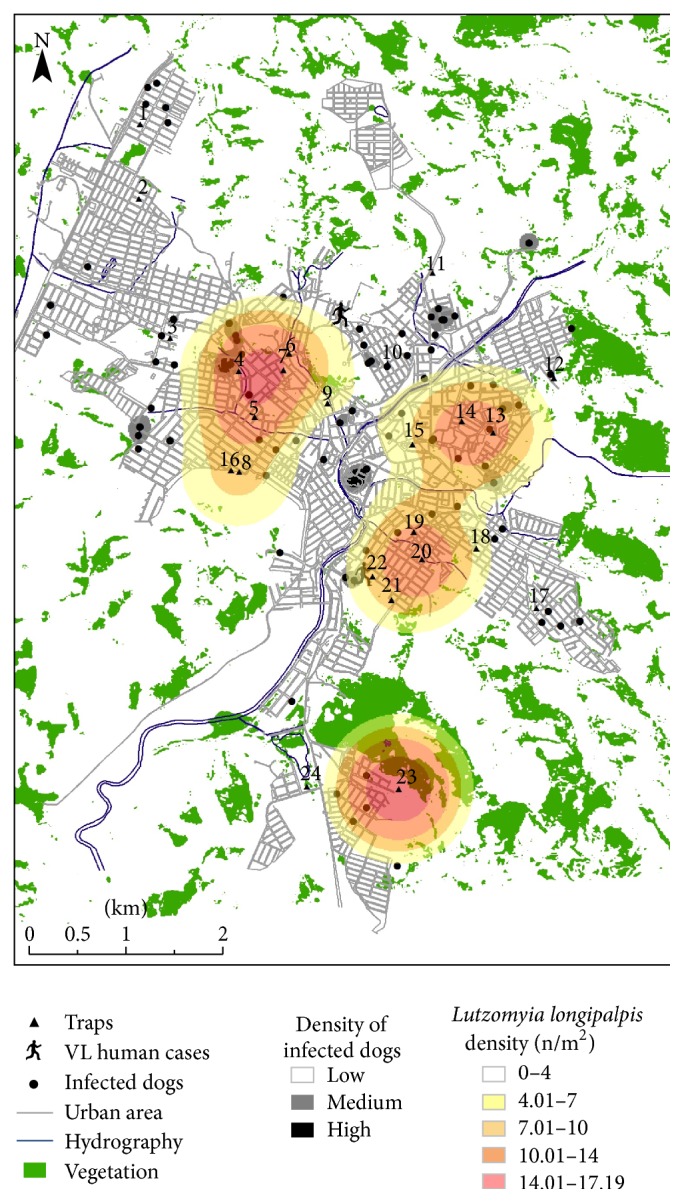
Geoprocessing analysis of captured* L. longipalpis* and parameters studied. Kernel density map of captured* L. longipalpis*, canine VL cases, and human VL cases (human icon) and representation of vegetation and hydrographic variables in the urban area of Formiga, Minas Gerais, Brazil. Dark circles indicate positive dogs either by serological or molecular methods. n, number of collected sand flies.

**Figure 7:**
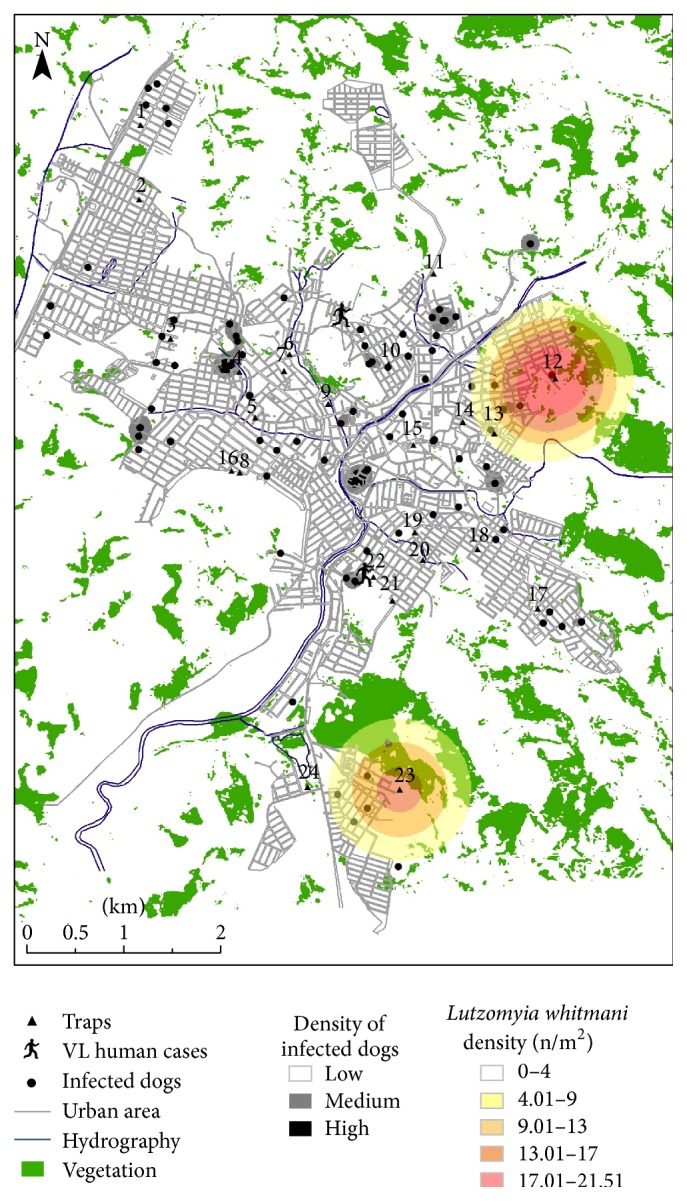
Geoprocessing analysis of captured* L. whitmani* and parameters studied. Kernel density map of captured* L. whitmani*, canine VL cases, and human VL cases (human icon) and representation of vegetation and hydrographic variables in the urban area of Formiga, Minas Gerais, Brazil. Dark circles indicate positive dogs either by serological or molecular methods. n, number of collected sand flies.

Based on the spatial analysis, higher sand fly densities were observed in the south, northeast, and northwest regions of the city (Figure 5). Also, a wider distribution of* L. longipalpis* (Figure 6) compared to* L. whitmani* (Figure 7) was found. On the other hand, positive dogs exhibited a punctual distribution in the city (Figures 5, 6, and 7, gray and black spots). Most of the sand flies were observed within a distance of 200 m from the vegetation, especially for* L. whitmani* (99.8%) and* L. longipalpis* (94.2%). However, the latter was also observed in areas up to 400 m from the vegetation. Similarly to the sand flies, a higher density of positive dogs (91.8% and 99.1%) was also located within 200 m from the vegetation areas and water collections, respectively (Figure 5).* Lutzomyia longipalpis* was captured up to 600 m from the water with higher densities within 400 m. Interestingly, overlapping distributions of sand flies and dogs indicated that 39.5% of the positive animals were found in areas with high insect densities (Figure 5). Finally, signature analysis did not show any relationship between dogs and* L. whitmani* and this was also observed in Figure 7.

Regarding VL human cases, those occurred in areas with low densities of* L. longipalpis* (0–4 insects/m^2^) (Figure 6). Similarly, low densities of infected dogs were observed where human cases were detected (Figure 5). However, signature analysis together with Figure 5 shows a slight overlapping of those variables in the areas of low vector and dog densities.

## 4. Discussion

The phenomenon of VL urbanization has been increasingly reported in many cities of Brazil [5–8]. For this reason, the present work had the objective of elucidating some epidemiological aspects of VL in Formiga, an area considered of recent transmission. Those included human and canine cases and sand flies. More importantly, spatial analysis mapped the critical areas for control interventions and surveillance.

Many proven and suspected vectors of either VL or CL were found in the city of Formiga. However, they were in lower densities when compared to other studies at nearby locations such as Divinópolis and Belo Horizonte [12, 15, 16]. Nevertheless, the existence of vectorial transmission cannot be discharged and is in agreement with the low number of human cases reported in the area. Consistent with those observations, even in places with low density, the potential of the sand flies to transmit the disease is enough for the maintenance of the parasite [38]. In spite of that,* L. longipalpis* was the most captured and dispersed sand fly, whose urban behavior was also confirmed in the city of Formiga. This may be an alert for the health authorities for the risk of a VL outbreak. Although no human CL cases were reported, the presence of* L. whitmani* in higher densities and the detection of* L. braziliensis* in the dogs demonstrated that the region may be susceptible to the disease. Although the role of domestic dogs as reservoirs of this species was not completely elucidated [39], the molecular data demonstrated that it is circulating in the area together with* L. infantum*.

In Brazil, the main strategy for VL control has been based on the elimination of seropositive dogs, although this is not widely accepted. This is recommended especially in areas where the seroprevalence is above 2%. In this study, the frequency of positive dogs using the two recommended serological methods (DPP and ELISA) was 5.8%. Studies of the canine seroprevalence in other urban areas showed similar results, although they did not use the same serological tests [8, 11, 27]. Another important fact to be considered is that a significant proportion of the seropositive dogs (38%) was subclinically infected (asymptomatic). However, they may be still infective for sand flies acting as urban reservoirs of the disease [40–43].

Many studies have shown that sand fly densities are influenced by climatic conditions especially high temperatures and humidity [5, 22, 44, 45]. Consistent with those observations, those factors also influenced the occurrence of sand flies, mainly in the summer months (January and February) when temperatures and precipitation increased. More importantly, the presence of vegetation is considered a risk factor for both VL and CL [46–48]. In this context, our spatial analyses have detected that* L. whitmani*,* L. longipalpis*, and positive dogs were closely related to the vegetation areas. Interestingly,* L. whitmani* was associated with the most forested areas in the city (Figure 7), a well described behavior for this species [15, 16, 36, 49–51]. Also, deforestation is an important factor for the dispersion of the species and appearance of CL cases [52].

On the other hand,* L. longipalpis* was more dispersed towards the urban areas. This is in agreement with the high adaptation to the artificial environments by this vector, even in areas with low vegetation [25, 53–55]. For this reason, health authorities should focus in spraying insecticides and monitor closely the infested areas by* L. longipalpis* in Formiga, avoiding its expansion towards other regions.

Most of the infected dogs were found at 200 m from the water, and this was a factor that may have influenced the distribution of canine cases in the city. Although rare in the literature, studies have demonstrated that proximity to water was a high risk factor for canine infection in Greece [56] and Spain [57]. Consistent with those observations, many reports have also associated that humidity and soil organic components promote sand fly development close to the water collections [36, 54].

Geoprocessing tools have been successfully used to monitor the geographic patterns involved in leishmaniasis transmission [58–60]. In this sense, many studies in different cities have associated epidemiological aspects with bioclimatic factors helping to define the most critical areas for control interventions [22, 47, 61]. This approach was also performed in Formiga and aimed at a better understanding of the VL transmission dynamics in the city. A spatial correlation demonstrating the simultaneous occurrence of human, canine, and proven vectors especially in the south, northeast, and northwest regions was observed. As the human cases are still very low when compared to endemic cities of the Minas Gerais state [8, 10, 13], control measures at this point may help to avoid human transmission and decrease canine infection.

## 5. Conclusions

Based on our findings, Formiga can be considered an area of recent transmission for VL and is on the risk for CL. Both vectors were found in the city and* L. longipalpis* was more dispersed than* L. whitmani*, being closely related to human and canine cases after spatial analysis. Molecular analyses have detected for the first time the presence of either* L. infantum* or* L. braziliensis* circulating in the domestic hosts. Those data reinforce the need of initial control measures for leishmaniasis in order to avoid expansion and future outbreaks of the disease in Formiga.

## Figures and Tables

**Table 1:** Frequency and percentage of phlebotomine sandflies captured from May/2012 to April/2013 using HP light traps in the municipality of Formiga, Minas Gerais state, Brazil.

Species	Sex		Total (%)
	M	F	
*Lutzomyia longipalpis *	63	16	79 (35.3)
*Lutzomyia cortelezzii *	38	37	75 (33.5)
*Lutzomyia whitmani *	36	5	41 (18.3)
*Lutzomyia lenti *	11	7	18 (7.9)
*Lutzomyia sordellii *	1	3	4 (1.7)
*Lutzomyia monticola *	1	2	3 (1.3)
*Lutzomyia lutziana *	0	1	1 (0.4)
*Lutzomyia bacula *	0	1	1 (0.4)
*Lutzomyia brasiliensis *	1	0	1 (0.4)
*Lutzomyia termitophila *	0	1	1 (0.4)

Total (%)	156 (68.1)	73 (31.9)	224 (100.0)
